# Supplementary material for: Sulforaphane Potentiates Gemcitabine-Mediated Anti-Cancer Effects against Intrahepatic Cholangiocarcinoma by Inhibiting HDAC Activity
Source: Cells. 2023 Feb 22;12(5):687. doi: 10.3390/cells12050687 (PMC10000472; doi:10.3390/cells12050687)
Supplement: Supplementary file 1 [file cells-12-00687-s001.zip › cells-2221123-supplementary.pdf]

cells

MDPI

Article

Sulforaphane Potentiates Gemcitabine-Mediated Anti-Cancer Effects against Intrahepatic Cholangiocarcinoma by Inhibiting HDAC Activity

Fumimasa Tomooka, Kosuke Kaji *, Norihisa Nishimura, Takahiro Kubo, Satoshi Iwai, Akihiko Shibamoto, Junya Suzuki, Koh Kitagawa, Tadashi Namisaki, Takemi Akahane, Akira Mitoro and Hitoshi Yoshiji

Department of Gastroenterology, Nara Medical University, Kashihara, Nara 634-8521, Japan
* Correspondence: kajik@naramed-u.ac.jp; Tel.: +81-744-22-3051; Fax: +81-744-24-7122

Supplementary Materials

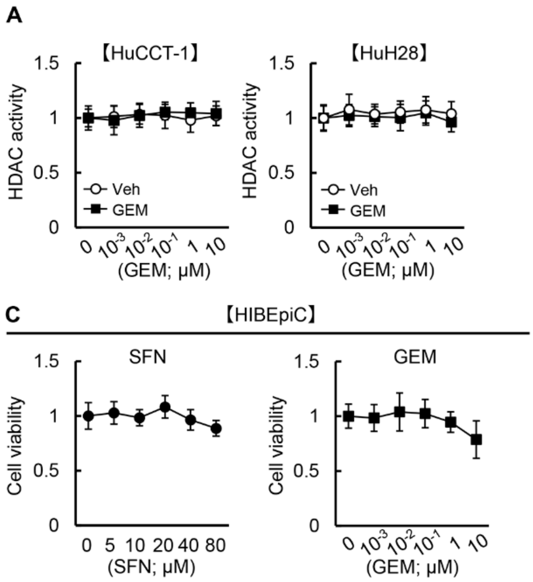

Figure S1. HDAC activity and cell viability in normal biliary epithelial cells. (A) HDAC activity in HuCCT-1 and HuH28 cells exposed to GEM (0–10 μM). (B) HDAC activity in normal HIBEpiC cells exposed to SFN (0–80 μM). (C) Cell viability in HIBEpiC cells exposed to SFN (0–80 μM) or GEM (0–10 μM). The values are shown as fold changes relative to 0 μM for each treatment group. Data are mean ± SD (n=3 independent experiments with n=8 samples per condition).

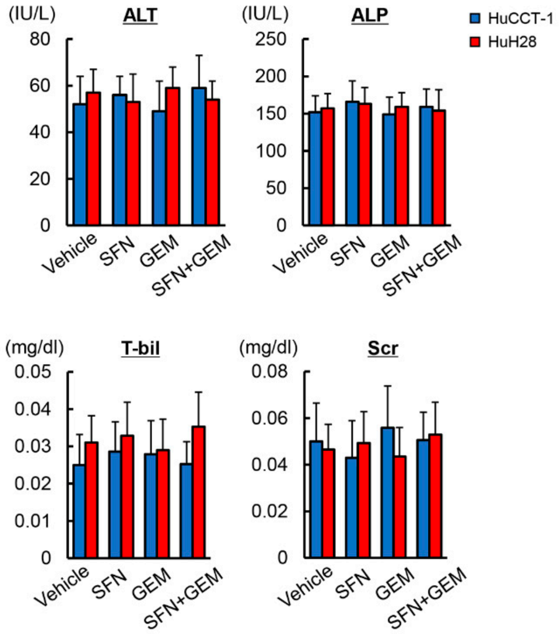

Figure S2. Effect of SFN on hepatic expression of anti-oxidative genes. (A) Relative mRNA expression of anti-oxidative genes (Hmox1, Nqo1, and Gstm3) in the liver of mice treated with different doses of SFN (0, 10, 25, 50 and 100 mg/kg/day) with concomitant GEM treatment (100 mg/kg twice a week). *Gapdh* was used as internal control for qRT-PCR. The values are shown as fold changes relative to SFN at 0 mg/kg/day group. Data are mean ± SD (n = 10). **p < 0.01 indicating a significant difference between groups.

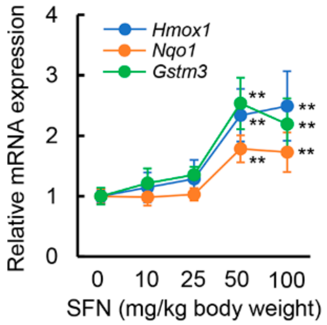

Figure S3. Liver, biliary and renal function in the HuCCT-1 and HuH28-xenografted mice. Serum levels of alanine aminotransferase (ALT), alkaline phosphatase (ALP), total bilirubin (T-bil), and creatinine (Scr) in the HuCCT-1 and HuH28-xenografted mice at the end of the experiment.

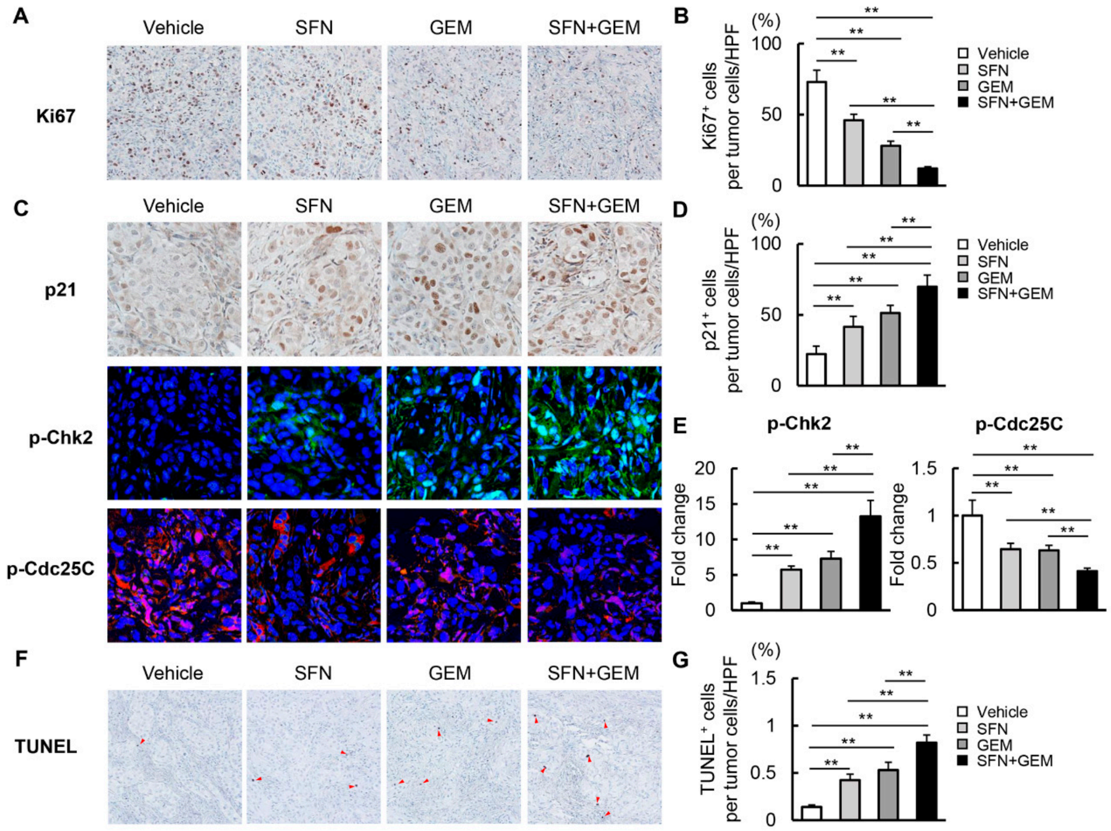

Figure S4. Cell proliferation and apoptosis in iCCA-derived xenograft tumors by treatment with SFN and GEM. (A, C, F) Representative images of HuH28-grafted tumors stained with Ki67 (A), p21, p-Chk2, and p-Cdc25C (C), TUNEL (F). Red triangles indicate intratumor apoptotic cells. Scale bar; 100 µm. (B) Quantification of Ki67+ proliferative cancer cells. The values are indicated as Ki67+ cancer cells/total cancer cells (%) in high power field (HPF). (D) Quantification of p21+ cancer cells. Quantitative values are indicated as p21+ cancer cells/total cancer cells (%) in HPF. (E) Semi-quantitation of p-Chk2+ or p-CdC25C+ cancer cells in HPF. The values are shown as fold changes relative to vehicle-treated group. (G) Quantification of TUNEL+ apoptotic cancer cells. The values are indicated as TUNEL+ cancer cells/total cancer cells (%) in HPF. Each quantitative analysis was performed for 10 fields per section. Data are mean ± SD (n=20 tumors/10 mice; B, D, E and G). * $p < 0.05$, ** $p < 0.01$, indicating a significant difference between groups. cells (%) in HPF. Each quantitative analysis was performed for 10 fields per section. Data are mean ± SD (n=20 tumors/10 mice; B, D, E and G). * $p < 0.05$, ** $p < 0.01$, indicating a significant difference between groups.

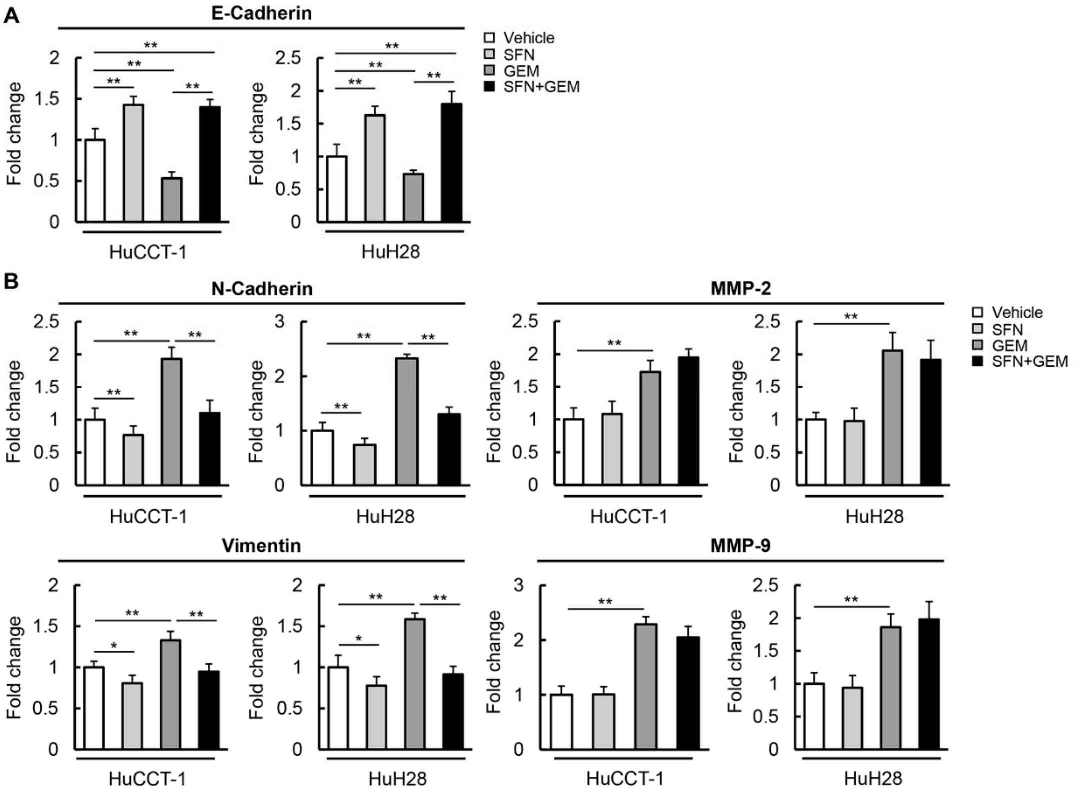

Figure S5. Protein levels of EMT-related markers in human iCCA-derived xenograft tumors. (A) Intratumor levels of E-Cadherin. (B) Intratumor levels of N-Cadherin, Vimentin, MMP-2 and MMP-9. Protein levels were determined using each ELISA assay. Quantitative values are relatively indicated as fold changes to the values of the vehicle-treated group. Data are mean ± SD (n=20 tumors/10 mice). * $p < 0.05$, ** $p < 0.01$, indicating a significant difference between groups.

Table S1. List of primers for quantitative RT-PCR.

Gene	Sense (5′-3′)	Antisense (5′-3′)
<u>Human</u>		
GAPDH	GTCTCCTCTGACTTCAACAGCG	ACCACCCTGTTGCTGTAGCCAA
CDKN1A	AGGTGGACCTGGAGACTCTCAG	TCCTCTTGGAGAAGATCAGCCG
BAX	TCAGGATGCGTCCACCAAGAAG	TGTGTCCACGGCGGCAATCATC
CDH1	AAGGCCTCTACGGTTTCATAACC	CACTGTATTCAGCGTGACTTTGG
KRT19	CTGAAGGAAGAGCTGGCCTA	TCATATTGGCTTCGCATGTC
VEGFA	CAGGCTGCTGTAACGATGAA	TTTCTTGCGCTTTCGTTTTT
VEGFR2	CAAGTGGCTAAGGGCATGGA	ATTTCAAAGGGAGGCGAGCA
HIF1A	CTCAAAGTCGGACAGCCTCA	CCCTGCAGTAGGTTTCTGCT
NOS3	GTGATGGCGAAGCGAGTGAAG	CCGAGCCCGAACACACAGAAC
VIM	AGTCCACTGAGTACCGGAGAC	CATTTCACGCATCTGGCGTTC
CDH2	GGCGTTATGTGTGTATCTTCACT	GCTTCTCACGGCATACACCA
MMP2	AGGGCACATCCTATGACAGC	ATTTGTTGCCCAGGAAAGTG
MMP9	CATCGTCATCCAGTTTGGTG	TCGAAGATGAAGGGGAAGTG
<u>Mouse</u>		
Hmox1	AACAAGCAGAACCCAGTCTATGC	AGGTAGCGGGTATATGCGTGGGCC
Nqo1	CAAGTTTGGCCTCTCTGTGG	AAGCTGCGTCTAACTATATGT
Gstm3	CCCCAACTTTGACCGAAGC	GGTGTCCATAACTTGGTTCTCCA
Vegfa	CTGCTGTAACGATGAAGCCCTG	GCTGTAGGAAGCTCATCTCTCC

Vegfr2	CGAGACCATTGAAGTGACTTGCC	TTCCTCACCCTGCGGATAGTCA
Cdh1	GGTCATCAGTGTGCTCACCTCT	GCTGTTGTGCTCAAGCCTTCAC
Krt19	AATGGCGAGCTGGAGGTGAAGA	CTTGGAGTTGTCAATGGTGGCAC
Cdh2	CCTCCAGAGTTTACTGCCATGAC	CCACCACTGATTCTGTATGCCG
Vim	CGGAAAGTGGAATCCTTGCAGG	AGCAGTGAGGTCAGGCTTGGAA
Mmp2	CAAGGATGGACTCCTGGCACAT	TACTCGCCATCAGCGTTCCCAT
Mmp9	GCTGACTACGATAAGGACGGCA	TAGTGGTGCAGGCAGAGTAGGA
Gapdh	CTGCGACTTCAACAGCAACT	GAGTTGGGATAGGGCCTCTC

Supplementary materials and methods

Cell proliferation assay

HuCCT-1 and HuH28 cells were seeded in 96-well plates with RPMI-1640 as previously described. Then, the cells were exposed to SFN and/or GEM at the IC_{50} for 24 or 48 h. DMSO was used as a vehicle. BrdU Cell Proliferation ELISA (Cosmo Bio, Tokyo, Japan) was used to evaluate cell proliferation according to the manufacturer's protocol. The proliferation rate was determined relative to that in the vehicle treatment group.

Flow cytometry

Flow cytometry was used for cell cycle analysis. HuCCT-1 and HuH28 cells were treated with SFN and/or GEM at the IC_{50} for 12 h. The cells were trypsinized and pelleted to a count of 1×10^6 cells. The cell pellet was washed twice with phosphate-buffered saline and agitated with 1 mL of hypotonic propidium iodide solution with the following components dissolved in distilled water: 50 µg/ml propidium iodide (Merck, Darmstadt, Germany), 0.1% trisodium citrate dihydrate (FUJIFILM Wako Pure Chemical Corporation, Osaka, Japan), 0.2% NP-40 ALTERNATIVE (Merck), and 0.25 mg/mL ribonuclease A from bovine pancreas (Merck) [1]. The cell suspensions were incubated at 4°C for 30 min in the dark and then incubated at 37°C for 20 min in the dark. The cell suspensions were agitated and analyzed using a Spectral Cell Analyzer SA3800 (Sony, Tokyo, Japan).

Western blotting

HuCCT-1 and HuH28 cells were treated with SFN and/or GEM at the IC_{50} for 12 h. Harvested protein extracts from both cell lines were prepared in T-PER Tissue Protein Extraction Reagent containing

proteinase and phosphatase inhibitors (Thermo Fisher Scientific, Rockford, IL, USA). The protein content was measured using a Takara BCA Protein Assay Kit (Takara Bio, Kusatsu, Japan). In total, 100 μg of whole cell lysates were separated by sodium dodecyl sulfate-polyacrylamide gel electrophoresis (NuPAGE; Thermo Fisher Scientific) and transferred to Invitrolon polyvinylidene difluoride membranes (Thermo Fisher Scientific), which were subsequently blocked for 1 h with 5% bovine serum albumin in Tris-buffered saline supplemented with Tween-20. Thereafter, the membranes were incubated overnight at 4°C with rabbit anti-p21 polyclonal antibody (Abcam, Cambridge, UK; cat: ab227443, 1:1000), rabbit anti-phospho-Chk2 (Thr68) (C13C1) monoclonal antibody (Cell Signaling Technology, Danvers, MA, USA; cat: #2197, 1:1000), rabbit anti-phospho-Cdc25c (Ser216) (63F9) monoclonal antibody (Cell Signaling Technology; cat: #4901, 1:1000), rabbit anti-Chk2 (D9C6) monoclonal antibody (Cell Signaling Technology; cat: #6334, 1:1000), rabbit anti-Cdc25c (5H9) monoclonal antibody (Cell Signaling Technology; cat: #4688, 1:1000), rabbit anti-BAX (D2E11) monoclonal antibody (Cell Signaling Technology; cat: #5023, 1:1000), rabbit anti-Bcl-2 (D17C4) monoclonal antibody (Cell Signaling Technology; cat: #3408, 1:1000), and rabbit anti-β-actin polyclonal antibody (Cell Signaling Technology; cat: #4967, 1:1000). The membranes were washed and incubated for 1 h with Goat Anti-Rabbit IgG H&L (HRP) (Abcam; 1:5000) and developed using Clarity Western enhanced chemiluminescence substrate (Bio-Rad, Hercules, CA, USA). Immunoblotting bands were densitometrically analyzed using ImageJ 64-bit Java 1.8.0 (National Institutes of Health, Bethesda, MA, USA).

Measurement of cleaved caspase-3

HuCCT-1 and HuH28 cells were exposed to SFN and/or GEM at the IC_{50} for 12 h. To examine *in vitro* cell apoptosis, cleaved caspase-3 concentrations in HuCCT-1 and HuH28 cell extracts were measured using a Human Cleaved Caspase-3 (Asp175) ELISA Kit (Abcam) according to the manufacturer's instructions.

Cell invasion and migration assay

Cytoselect 24-well cell invasion assay kit (Cell Biolabs, Inc.) and Cytoselect 24-well cell migration assay kit (Cell Biolabs, Inc., San Diego, CA) were used according to the manufacturer's protocol. Briefly, HuCCT-1 and HuH28 cells (1×10^6 cells/mL) were suspended in serum-free RPMI-1640 with SFN and/or GEM at

the IC_{50}. Then, 300 μl of cell suspension containing 1×10^6 cells/ml was added to the 8 μm pore size polycarbonate membrane cell culture inserts coated with a uniform layer of basement membrane matrix solution for cell invasion assay or to the 8 μm pore size polycarbonate membrane cell culture inserts for cell migration assay, respectively. Then, cells were incubated for 24 hr at 37°C in a 5% CO2 atmosphere. Next, the medium was removed from the inside of the insert and the interior of the insert was gently swabbed with the wet end of cotton-tipped swabs to remove non-migratory cells. Then, each insert was transferred to a clean well containing 400 μl of cell stain solution and incubated for 10 min at room temperature. Invasive cells were counted under a light microscope with at least three individual fields per insert observed. After washing, inserts were transferred to an empty well and 200 μl of extraction solution was added and incubated for 10 min. Then, the optical density (OD) was measured at 560 nm.

RNA isolation, cDNA synthesis, and real-time PCR

Total cellular RNA was isolated from cultured HuCCT-1 and HuH28 cells and resected subcutaneous tumor tissues using a RNeasy Mini Kit (Qiagen, Hilden, Germany) according to the manufacturer's instructions. cDNA was prepared with 2 μg of total RNA using a High-Capacity RNA-to-cDNA kit (Applied Biosystems, Foster City, CA, USA) according to the manufacturer's instructions. Real-time PCR was performed using the StepOnePlus Real-time PCR system and SYBR Green (Applied Biosystems). The primer sequences are presented in Table S1. Gene expression was normalized to that of the housekeeping gene Glyceraldehyde 3-phosphate dehydrogenase (GAPDH). mRNA levels were calculated using the $2^{-\Delta\Delta Ct}$ method and expressed as fold changes relative to the control level in each experiment [2].

Histological and immunohistochemical analyses

Resected subcutaneous tumor specimens were fixed in 10% formalin, incubated overnight at room temperature, and embedded in paraffin. Then, 5-μm-thick liver sections were stained with hematoxylin and eosin (H&E) (Narabyouri Research Co., Nara, Japan). iCCA cell apoptosis was determined by the TUNEL assay using an In situ Apoptosis Detection Kit (Takara Bio) on paraffin-embedded tumor sections as recommended by the supplier. The sections were counterstained with hematoxylin.

Immunohistochemical and immunofluorescence analyses were performed using paraffin-embedded tumor sections. The sections were pre-treated with heat-mediated antigen retrieval with sodium citrate buffer (pH 6.0) for 20 min. For immunohistochemistry, the sections were incubated at 4°C overnight with rabbit anti-Ki67 polyclonal antibody (Abcam; cat: ab15580, 1:100), rabbit anti-p21 polyclonal antibody (Abcam; cat: ab227443, 1:1000), or rabbit anti-CD34 (EP373Y) monoclonal antibody (Abcam; cat: ab81289, 1:2500) as the primary antibody. Thereafter, the sections were washed and incubated with a goat anti-rabbit biotinylated secondary antibody (Vector Laboratories, Burlingame, CA, USA; 1:200) to detect the primary antibodies and visualized using a horseradish peroxidase-conjugated ABC system (Vector Laboratories). DAB was used as the chromogen. For immunofluorescence, rabbit anti-phospho-Chk2 (Thr68) (C13C1) monoclonal antibody (CST; cat: #2197, 1:100) and rabbit anti-phospho-Cdc25c (Ser216) (63F9) monoclonal antibody (CST; cat: #4901, 1:100) were used as the primary antibodies. The primary antibodies were detected using Alexa Fluor-conjugated secondary antibodies (Invitrogen, Waltham, MA, USA); subsequently, nuclear staining was performed using 4′,6-diamidino-2-phenylindole Fluoromount-G mounting medium (Vector Laboratories). Images were captured using a BX53 microscope (Olympus, Tokyo, Japan) for histology and immunohistochemistry and an FV3000 microscope (Olympus) for immunofluorescence. Semi-quantitative analysis was performed for 10 fields per section at ×400 magnification using ImageJ 64-bit Java 1.8.0.

Experimental protocol for in vivo titration of sulforaphane

Fifty of ten-week-old male C57BL/6J mice (CLEA Japan, Osaka, Japan) were divided into five groups (n = 10 mice/group) and received oral administration of different doses of SFN (0, 10, 25, 50 and 100 mg/kg/day) with concomitant GEM treatment (100 mg/kg twice a week) for each group. All mice were housed in stainless steel mesh cages under controlled conditions (23°C ± 3°C with a relative humidity of 50% ± 20%, 10–15 air changes/hour, and 12 hours of light/day). All animals were allowed *ad libitum* access to tap water throughout the experimental period. All mice were sacrificed after four weeks of feeding. At the end of the experiments, all mice underwent the following procedures: anesthesia with barbiturate overdose (intravenous injection, 150 mg/kg pentobarbital sodium), opening of the abdominal cavity, blood collection via aortic puncture, and harvesting of liver. All animal procedures were performed

in compliance with the recommendations of the Guide for Care and Use of Laboratory Animals of the National Research Council, and the study was approved by the Animal Care Committee of Nara Medical University.

Enzyme-Linked Immunosorbent Assay (ELISA) assay for intratumor levels of EMT-related markers

Protein tissue lysates were prepared from 20 mg of frozen subcutaneous tumor tissue using T-PER Tissue Protein Extraction Reagent containing proteinase and phosphatase inhibitors (Thermo Fisher Scientific). The protein content was measured using a Takara BCA Protein Assay Kit (Takara Bio). Intratumor levels of E-Cadherin, N-Cadherin, Vimentin, MMP-2 and MMP-9 were determined using Human E-Cadherin ELISA Kit (Abcam), Human N-Cadherin ELISA Kit (Abcam), Human Vimentin ELISA Kit (Abcam), Human MMP2 ELISA Kit (Abcam), Human MMP9 ELISA Kit (Abcam), respectively, according to the manufacturer's instructions.

References

1. Deitch, A.D.; Law, H.; White, R.D. A stable propidium iodide staining procedure for flow cytometry. *J. Histochem. Cytochem.* **1982**, *30*, 967–972.
2. Livak, K.J.; Schmittgen, T.D. Analysis of relative gene expression data using real-time quantitative PCR and the 2(-Delta Delta C(T)) Method. *Methods* **2001**, *25*, 402–408.
